# Bullseye's representation of cerebral white matter hyperintensities

**DOI:** 10.1016/j.neurad.2017.10.001

**Published:** 2018-03

**Authors:** C.H. Sudre, B. Gomez Anson, I. Davagnanam, A. Schmitt, A.F. Mendelson, F. Prados, L. Smith, D. Atkinson, A.D. Hughes, N. Chaturvedi, M.J. Cardoso, F. Barkhof, H.R. Jaeger, S. Ourselin

**Affiliations:** a Translational Imaging Group, CMIC, Department of Medical Physics and Biomedical Engineering, University College London, Room 8.04 8th floor Malet Place Engineering Building, 2, Malet Place, WC1E 7JE London, UK; b Dementia Research Centre, UCL Institute of Neurology, WC1N 3BG London, UK; c Santa Creu i Sant Pau Hospital, Universitat Autonòma Barcelona, 08041 Barcelona, Spain; d Lysholm Department of Neuroradiology, The National Hospital for Neurology and Neurosurgery, Queen Square, WCN1 3BG London, UK; e Brain Repair and Rehabilitation, UCL Institute of Neurology, WC1N 3BG London, UK; f Cardiometabolic Phenotyping Group, UCL Institute of Cardiovascular Science, W1CE 6HX London, UK; g Centre for Medical Imaging, UCL Faculty of Medical Science, NW1 2PG London, UK

**Keywords:** White matter hyper intensities, Visual rating scales, Magnetic resonance imaging, Location, Ageing, BG, basal ganglia, CI, confidence Interval, FLAIR, fluid attenuated inversion recovery, ICC, intraclass correlation, IQR, interquartile range, IT, infratentorial regions, JC, juxtacortical, K_τ_, Kendall's tau, MR, magnetic resonance, PV, periventricular, SD, standard deviation, WMH, white matter hyperintensities

## Abstract

**Background and purpose:**

Visual rating scales have limited capacities to depict the regional distribution of cerebral white matter hyperintensities (WMH). We present a regional-zonal volumetric analysis alongside a visualization tool to compare and deconstruct visual rating scales.

**Materials and methods:**

3D T1-weighted, T2-weighted spin-echo and FLAIR images were acquired on a 3 T system, from 82 elderly participants in a population-based study. Images were automatically segmented for WMH. Lobar boundaries and distance to ventricular surface were used to define white matter regions. Regional-zonal WMH loads were displayed using bullseye plots. Four raters assessed all images applying three scales. Correlations between visual scales and regional WMH as well as inter and intra-rater variability were assessed. A multinomial ordinal regression model was used to predict scores based on regional volumes and global WMH burdens.

**Results:**

On average, the bullseye plot depicted a right-left symmetry in the distribution and concentration of damage in the periventricular zone, especially in frontal regions. WMH loads correlated well with the average visual rating scores (e.g. Kendall's tau [Volume, Scheltens] = 0.59 CI = [0.53 0.62]). Local correlations allowed comparison of loading patterns between scales and between raters. Regional measurements had more predictive power than global WMH burden (e.g. frontal caps prediction with local features: ICC = 0.67 CI = [0.53 0.77], global volume = 0.50 CI = [0.32 0.65], intra-rater = 0.44 CI = [0.23 0.60]).

**Conclusion:**

Regional-zonal representation of WMH burden highlights similarities and differences between visual rating scales and raters. The bullseye infographic tool provides a simple visual representation of regional lesion load that can be used for rater calibration and training.

## Introduction

White manner hyperintensities (WMH) in the cerebral white matter on T2-weighted spin echo and FLAIR magnetic resonance (MR) images are commonly part of the spectrum of imaging findings in cerebral small vessel disease and normal aging. However, their precise etiology is still a subject of debate and likely multifactorial [1]. Histological findings in WMH include thinning or disruption of the myelin sheath, axonal loss and gliosis [2]. Close to the ventricles, increased water content in the extracellular spaces has been reported when the ependymal lining is damaged [2]. WMH are very prevalent and are associated with various clinical symptoms such as a decreased processing speed, altered gait, incontinence and depression [3]. Studies have demonstrated a link between the burden of WMH and cortical blood flow [4] as well as with cardiovascular risk factors such as hypertension [5] or diabetes [6]. In addition, the extent of WMH was recently shown to be an independent risk factor for periprocedural stroke in patients undergoing stenting of a carotid artery stenosis [7] and an indicator of prognostic outcome after ischemic stroke [8].

The majority of studies relating clinical findings with the burden of WMH have used visual rating scales. Such scales provide a semi-quantitative way to describe the burden and distribution of WMH in the brain without manual lesion delineation, a task that is cumbersome, time consuming and subject to inter- and intra-rater variability. A number of visual rating scales with various levels of complexity have been developed [9], [10], [11], [12], [13], [14]. Compared to automatic global volumetric assessments, they remain popular especially when incorporating local burden information. The spatial information of WMH distribution, incorporated in the rating scales ranges from whole brain assessment (Manolio [9], simplified Fazekas [15]) to specific lobar lesion burden (Scheltens [16]). While spatial determination allows for differential clinical and pathophysiological explanatory pathways, the definition of the regional borders can be ambiguous and varies from one scale to another. With respect to the separation of periventricular and deep WMH, most methods are based on absolute distance to the ventricles and do not take into account additional age-related changes such as ventricular expansion [17]. Finally, few scales have been specifically defined for the longitudinal assessment of the WMH burden, whereas most are only intended to be applied cross-sectionally [18].

With the recent advances in the automated identification of WMH, lesion volume has been shown to be associated with clinical outcomes, sometimes allowing for a better differentiation between clinical subgroups than visual rating scales [19]. The correlation between visual scales is considerable [20] but heterogeneity between visual rating systems has also been put forward as a potential explanation for contradictory findings [21]. Methods involving the creation of voxelwise lesion maps have been proposed to investigate WMH spatial distribution across populations [22] or in relation to specific risk factors [23]. These strategies suffer however from a high noise level due to the sparsity of the lesions. In contrast, region based strategies generally consider a separation between zones based on the absolute distance to the ventricles and thus cannot account for the variability in atrophy across subjects [24].

This work presents a novel approach to analyze regional-zonal WMH burden. We used it to deconstruct the spatial loading of visual rating scales and determine in an objective manner similarities and discrepancies between such scales, but also to formally address interobserver variability. The bullseye infographic provides a simple visual tool to train raters or display disease effects.

## Material and methods

### Cohort imaging study

We used an imaging data subset of the SABRE study (UK Clinical Trials Gateway DRN 841, local ethical approval by Fulham REC ref: 14/LO/0108) comprising the first 84 consecutive participants a tri-ethnic population based study [mean (SD) age = 71.4 (5.7) years; 61.7% male]. This cohort study aims to assess the risks of diabetes and cardiovascular disease, including small vessel disease in the brain, in European, Indian Asian and African Caribbean men and women [25]. Surviving participants of 4972 individuals recruited in 1988–1990 from general practices in the London boroughs of Southall and Brent were all invited for this third round of investigations. Spouses of the participants were also invited to take part. Participants were excluded from the study on clinical ground if they were at a stage of terminal illness or if severe comorbidities affected their attendance and/or participation to the investigations.

All participants gave informed written consent and underwent MRI according to a standard protocol on a Philips Achieva 3.0-Tesla scanner. Imaging included the following pulse-sequences:

• 3D sagittal T1-weighted FFE: TR 6.9 ms; TE 3.1 ms; voxel size 1.0 × 1.0 × 1.0 mm^3^;
• 3D sagittal T2-weighted FLAIR: TR 4800 ms; TI 1650 ms; TE 125 ms; voxel size 1.0 × 1.0 × 1.0 mm^3^;
• 3D sagittal T2-weighted TSE: TR 2500 ms TE 222 ms; voxel size 1.0 × 1.0 × 1.0 mm^3^.

All images were reviewed for incidental pathology and scan quality. Two participants’ scans were discarded from the analysis due to severe motion artifacts.

### Regional-zonal WMH burden quantification

WMH were automatically segmented using a previously developed algorithm [26]. In brief, this iterative model selection framework uses simultaneously the three MRI pulse sequences to model both normal and outlier observations as a multivariate Gaussian mixture informed by anatomical atlases and constrained to ensure neighborhood consistency. Once the data model is fitted, the actual lesion segmentation is performed by voxelwise comparison to normal appearing white matter.

A patient-specific coordinate frame was created to localize the WMH burden. This coordinate frame considered radially the relative distance between the ventricles and the cortical grey matter discretized into four equidistant layers. As described by Yezzi and Prince [27], this distance was derived from the solution to the Laplace equation applied here between the ventricular surface and the white matter/cortical gray matter interface. By design, such distance is made agnostic to the level of observed atrophy. A division of the white matter into lobes provided the angular information. The division into lobes was based on the Euclidean distance maps resulting from the cortical parcellation obtained through the application of a label-fusion method [28]. Frontal, parietal, temporal and occipital lobes were delineated on the right and left side, while the basal ganglia, thalami and infratentorial regions from both sides were combined (BGIT region). By combining the 4 layers and the 9 lobar zones, 36 regions were defined in total.

**Fig. 1:**
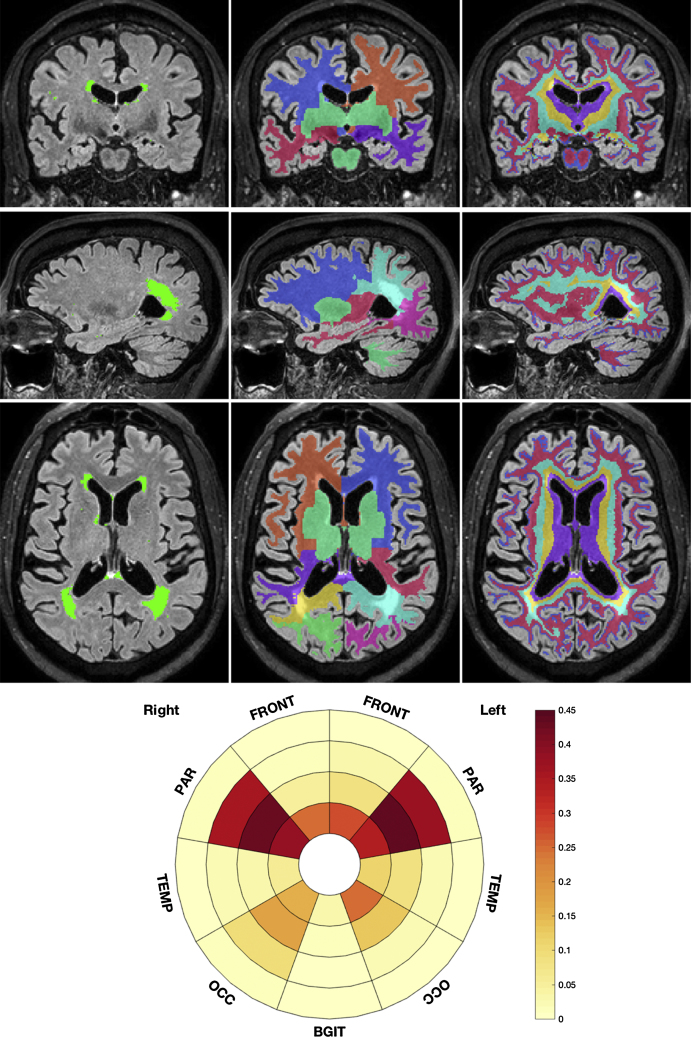
Representation of the building blocks of the local WMH lesion loads. The first column reflects the lesion segmentation. The second column refers to the separation according to the lobar regions and the last column to the distance based layer separation from the ventricular surface towards the cortical sheet. The lesion frequency per defined local region is then summarized in the bullseye plot. Most central parts correspond to the most periventricular regions. The lobar regions are represented according to the angular position and referred to by their first letters. The subject is male, 75 years old.

The proportion of each region affected by WMH was used as a local feature and is referred to as regional WMH load hereafter. Once the local quantitative values are extracted, they are summarized as an infographic in a bullseye plot: the 4 layers are represented concentrically, the closest to the center being the most periventricular. The lobes are referred to by their first letters (Front, Par, Occ, Temp, BGIT). Fig. 1 illustrates the definition of the regional WMH loads and their bullseye representation for a typical subject.

### Visual rating scales

The FLAIR scans were rated by four different raters with different levels of expertise (CHS 2y, BGA 23y, ID 10y, AS 3y). Each rater scored the scans according to three well-established visual rating scales that range from a global impression to more fine-grained regional scores [20]. The scales are summarized as follows:

• Manolio scale [29]: designed for the Cardiovascular Health study. The scale characterizes the WMH burden globally and ranges from 0 (absence) to 9 (highest degree) by matching to a template;
• Fazekas scale [15]: designed for aging subjects in a dementia study. The WMH rating is dichotomized between periventricular and deep WMH, assessed on a 4 point scale from 0 (absence) to 3 (highest degree) and a composite score is obtained by summing the subscales;
• Scheltens scale [16]: designed for aging subjects probably affected by Alzheimer's disease. The WMH rating is defined differently according to global regions: periventricular lesions (score range: 0–6), deep white matter per lobe (total score range: 0–24), basal ganglia per nucleus (total score range: 0–30) and infratentorial regions (score range: 0–24) themselves separated in subregions. Periventricular and deep regions are dichotomized based on the absolute distance (10 mm) to the ventricular surface.

### Statistical analysis

The scores given by the different raters were averaged to produce mean scores. The average scores were correlated with the automated regional WMH burden to illustrate the spatial correspondences between scores on the different scales and the frequency of WMH.

In a next step, the individual visual scores for each rater were correlated with the automated regional WMH loads. With the aim of studying the degree of consistency/bias between each rater and the average, the degree of regional interactions for each rater was compared to the degree of regional interactions of the average ratings.

The global WMH burden and scale-specific aggregate regional burden estimates were used as features to predict the rating scales. A multinomial ordinal regression model was used in a stratified 2-fold cross-validation procedure with 50 repeats. Predictions were obtained for the average of two, three or four raters. The ability to predict the rating scales was tested using either the global relative WMH burden or the scale-specific aggregate WMH loads.

Inter-rater variability was estimated as the average pairwise intraclass correlation (ICC) between raters. Intra-rater variability was estimated by the ICC of repeat measurements of one single rater on a subset of 20 subjects (2 measurements with a 6 months time interval).

## Results

### Population WMH distribution

**Fig. 2:**
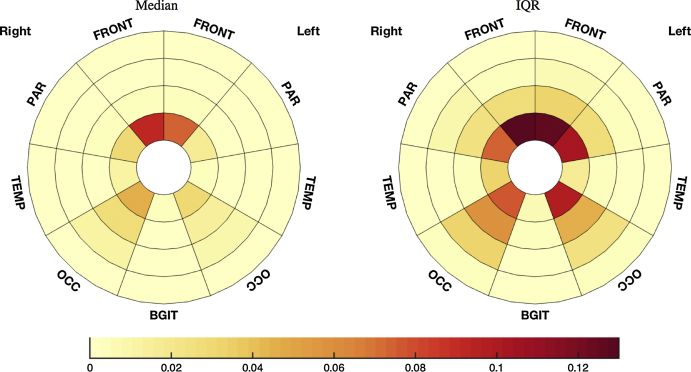
Median (left) and IQR (right) of the WMH burden frequency per zone represented in bullseye plot.

The extracted total WMH burden for the 82 subjects with available MR scans ranged from 0.38 mL to 25.28 mL (median 1.71 mL, IQR [0.81 mL 4.57 mL]). Fig. 2 represents the median WMH distribution across all subjects and the corresponding IQR. It illustrates the right-left symmetry as well as the prevalence of WMH in periventricular zones compared to deeper layers [30], the sparing of the infratentorial regions and the tendency towards greater WMH burdens in the frontal regions [31] described in the literature.

### Global comparison between volumes and visual scales

The Kendall's tau (Kτ) correlations between quantitative volumes and visual rating scales (global scores) across all raters are gathered in Table 1. All correlations were statistically significant with *P*-values < 0.0005 and only the correlation between Manolio and Fazekas was significantly higher than any other.

**Table 1:** Summary of Kendall's tau correlation results between global scale scores.

			Mean	SD	Min	Max	CI
Volume	–	Manolio	0.61	0.01	0.60	0.61	[0.57 0.64]
Volume	–	Fazekas	0.58	0.02	0.56	0.60	[0.54 0.61]
Volume	–	Scheltens	0.59	0.03	0.55	0.62	[0.55 0.62]
Manolio	–	Fazekas	0.72	0.02	0.71	0.75	[0.70 0.75]
Manolio	–	Scheltens	0.64	0.02	0.62	0.67	[0.60 0.67]
Fazekas	–	Scheltens	0.61	0.02	0.58	0.63	[0.57 0.65]

All correlations were statistically significant with *P*-values < 0.0005. There was no significant difference between the correlations except for the Manolio–Fazekas correlation that was significantly stronger than all the others.

In line with the literature [12], [32], there was a good agreement between the various scales. In addition, visual scales and WMH volumes were strongly correlated with Kendall's tau coefficients of 0.59 (CI = [0.53 0.62]), 0.58 (CI = [0.54 0.61]) and 0.61 (CI = [0.57 0.63]) for the Scheltens, the Manolio and the Fazekas scales respectively. The intra-rater ICC evaluated in a subset of 20 subjects were 0.70 (CI = [0.19 0.89], 0.68 (CI = [0.34 0.86], 0.70 (CI = [0.01 0.91] while the mean pairwise inter-rater ICC were 0.70 (CI = [0.26 0.86]) 0.80 (CI = [0.67 0.87] and 0.64 (CI = [0.38 0.79] for the Scheltens, Manolio and Fazekas scales respectively.

### Visual scale local deconstruction

**Fig. 3:**
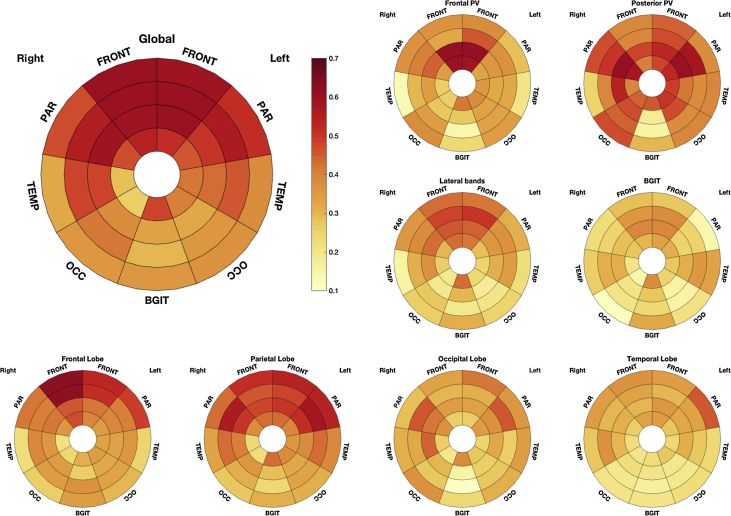
Kendall's tau correlation between the regional WMH lesion loads and each Scheltens subscale. See plot titles for the corresponding evaluated region. On the bottom row from left to right: frontal lobe, parietal lobe, occipital lobe and temporal lobe. Note the higher correlations between the periventricular subscales and central WMH loads in the bullseyes and at the periphery of the plot for lobar scores. The bigger plot on the left represents the correlations between the global score and the local lesion frequencies, showing that the frontal lobe had the highest overall loading.

Using a similar representation as the one used in Fig. 1, the correlations between the average Scheltens subscales and the regional descriptors are illustrated in Fig. 3.

The observed correlations were stronger for the subscales related to easily defined regions such as the frontal and posterior periventricular regions. Correlation patterns were in accordance with subscale definitions. For instance, the frontal periventricular (ScheltensFC) scale was significantly more correlated with the frontal most periventricular region (FPV) than with the frontal most juxtacortical (FJC) one (p-value < 0.01, K_τ_(FPV, ScheltensFC)–K_τ_ (FJC, ScheltensFC) = 0.23, CI = [0.19 0.28]). The clear difference in observed patterns when comparing the frontal lobe and the parietal lobe further supports the assumption that certain local features drive the visual rating process. Areas with a low probability of WMH (e.g. temporal lobe) were found to be less associated with any of the scales. Finally, a high degree of correlation was found across all regions when correlating with the Scheltens global scale.

### Interpreting raters’ behaviour

**Fig. 4:**
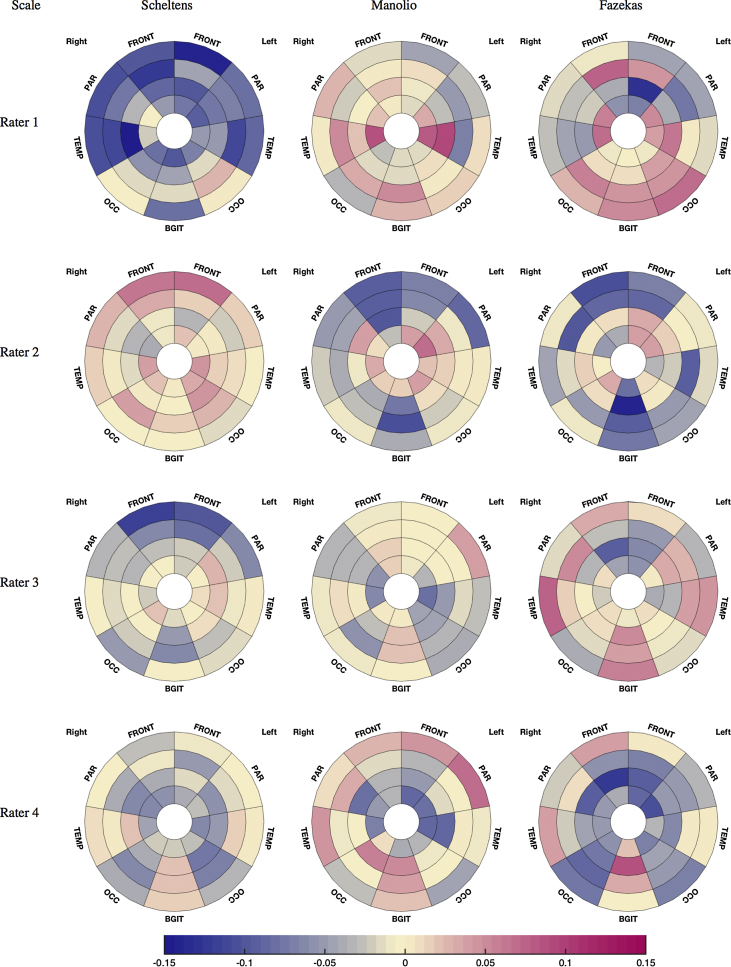
Plots of the rating discrepancies between one rater and the average of the others calculated as the difference between the Kendall's tau correlations of the local measures of WMH burden with one rater and with the average score given by the three remaining raters. Each column corresponds to a visual scale. Each row corresponds to a different individual rater.

For every scale, the correlation between each of the 36 automated local burden measures and the raters’ individual scores was calculated. Subsequently, the average scores for every possible combination of three raters was calculated in order to be compared with the individual scores of the fourth rater. Fig. 4 demonstrates the differences between the correlation obtained with one rater and with the average of the three remaining ones. In this figure, a pink color represents a numerically stronger and a blue color a numerically weaker interaction between a given rater's individual score and the regional lesion volume in comparison to the one found for the average score of the three other readers. Colloquially, this can be interpreted in the following way: the pink regions have relatively stronger influence on the individual rater's score, whereas the blue regions have a weaker influence. For example, in the Manolio scale grading, the influence of the three first layers of the parietal and frontal regions on rater #4's scores was lower than that of the average of the remaining raters, indicating that this rater could benefit from paying more attention to these areas when grading. However, the same rater appears to be comparatively more sensitive to WMH in the juxtacortical (4th layer) frontal and parietal regions.

### Local comparison between visual scales

**Fig. 5:**
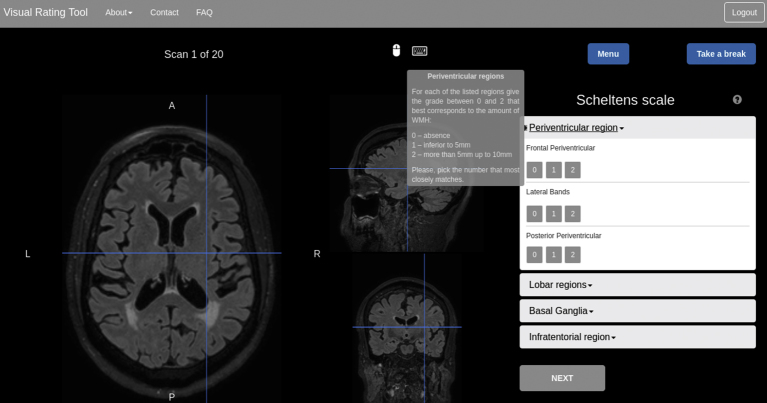
Plots of the correlations between local burden measures and the average of the four raters for each of the visual scales.

The correlations between local measures and the average of 4 raters are presented for each scale in Fig. 5. The three global scores show relatively similar patterns in the degree of regional loading, with a predominant effect of periventricular zones. Compared to both the Fazekas and the Manolio scales, the Scheltens scale appears to be more homogenously reflecting WMH loads across all brain regions. In particular, correlations with the juxtacortical regions (JC) are higher for the Scheltens than the Manolio and Fazekas scales, the difference reaching significance in both cases (K_τ_ (JC, Scheltens)–K_τ_ (JC, Manolio) = 0.036 CI = [0.004 0.068]; K_τ_ (JC, Scheltens)–K_τ_ (JC, Fazekas) = 0.11 CI = [0.07 0.15]). In turn, the Manolio scale presents highest loading by the periventricular regions (PV), the difference reaching significance when compared to the Fazekas scale (K_τ_ [PV, Manolio]–K_τ_ [PV, Fazekas]) = 0.11 CI = [0.06, 0.15].

### Explanatory power of local measurement

The ability to explain the local and global scales based on the consensus ratings is presented in Table 2. For all studied visual scales and subscales, the intraclass correlation between the predicted and the actual values when training on an average of 2, 3 or 4 raters and using either the designed local features or the global value were calculated. When appropriate (2 or 3 raters) the results are given under the form mean (SD). The correlations are compared to the average inter-rater ICC when correlating each rater with an average of complementary raters. Results show the following: firstly, when predicting subscales, the use of regional WMH burdens from the same anatomical location as the subscale allow for better predictions than using global features; secondly, the ability to predict the rating scale scores appears to increase with the number of raters used to establish the training average. The correlation between average scores and predictions, based on volumetric regional predictors was higher than the inter-rater variability for most scales, except in regions with a low prevalence of WMH (e.g. temporal lobe, BGIT – Fig. 3). For all subscales, the inter-rater correlation confidence interval was also found to be larger than for the automated prediction model.

**Table 2:** Explanatory value of the local WMH loads.

			Prediction using local features			Prediction using global volume			Raters		
			Pred4	Pred3	Pred2	Pred4	Pred3	Pred2	Ave3	Ave2	IR
Scheltens	PV	FC	**0.67**	**0.66**	**0.61**	0.50	**0.53**	0.48	0.53	0.51	0.44
			[0.53 0.77]	[0.51 0.76]	[0.45 0.73]	[0.32 0.65]	[0.36 0.67]	[0.29 0.63]	[0.30 0.69]	[0.29 0.67]	[0.23 0.60]
		LB	**0.46**	**0.43**	**0.38**	**0.43**	**0.41**	0.36	0.40	0.38	0.32
			[0.27 0.61]	[0.24 0.59]	[0.17 0.55]	[0.24 0.59]	[0.21 0.57]	[0.16 0.54]	[0.14 0.59]	[0.14 0.57]	[0.11 0.50]
		PC	**0.69**	**0.66**	**0.59**	**0.65**	**0.62**	**0.55**	0.43	0.40	0.33
			[0.56 0.79]	[0.53 0.77]	[0.43 0.71]	[0.51 0.76]	[0.47 0.74]	[0.38 0.68]	[0.21 0.60]	[0.19 0.57]	[0.13 0.51]
	Lobes	F	0.66	0.64	0.62	0.60	0.59	0.57	0.73	0.71	0.64
			[0.52 0.77]	[0.50 0.75]	[0.47 0.74]	[0.44 0.72]	[0.42 0.71]	[0.40 0.70]	[0.42 0.85]	[0.42 0.84]	[0.37 0.79]
		P	0.60	0.58	0.56	0.65	0.64	0.61	0.71	0.69	0.63
			[0.44 0.72]	[0.42 0.71]	[0.39 0.69]	[0.51 0.76]	[0.49 0.75]	[0.46 0.73]	[0.46 0.84]	[0.43 0.82]	[0.35 0.78]
		O	**0.55**	**0.46**	**0.37**	**0.47**	**0.42**	**0.35**	0.22	0.19	0.15
			[0.38 0.69]	[0.28 0.62]	[0.17 0.54]	[0.28 0.62]	[0.22 0.58]	[0.15 0.52]	[0.02 0.41]	[−0.02 0.39]	[−0.06 0.35]
		T	0.35	0.33	0.28	0.35	0.34	0.28	0.45	0.43	0.35
			[0.14 0.52]	[0.13 0.51]	[0.07 0.46]	[0.15 0.53]	[0.13 0.52]	[0.07 0.47]	[0.26 0.61]	[0.23 0.59]	[0.15 0.52]
		Partial Tot	**0.82**	**0.81**	**0.79**	**0.82**	**0.81**	**0.79**	0.76	0.75	0.69
			[0.73 0.88]	[0.72 0.87]	[0.69 0.86]	[0.73 0.88]	[0.73 0.88]	[0.70 0.86]	[0.27 0.90]	[0.34 0.89]	[0.26 0.85]
		BGIT	0.50	0.50	0.47	0.48	0.48	0.45	0.71	0.69	0.62
			[0.32 0.65]	[0.31 0.64]	[0.29 0.62]	[0.30 0.63]	[0.29 0.63]	[0.26 0.61]	[0.52 0.82]	[0.50 0.81]	[0.44 0.75]
		Tot	**0.83**	**0.82**	**0.80**	**0.83**	**0.82**	**0.81**	0.77	0.75	0.70
			[0.74 0.88]	[0.73 0.88]	[0.71 0.87]	[0.75 0.89]	[0.74 0.88]	[0.71 0.87]	[0.24 0.90]	[0.32 0.89]	[0.26 0.86]
		Manolio	0.84	0.84	0.82	0.83	0.83	0.81	0.86	0.84	0.80
			[0.76 0.89]	[0.76 0.89]	[0.73 0.88]	[0.75 0.89]	[0.75 0.89]	[0.72 0.87]	[0.76 0.91]	[0.74 0.90]	[0.67 0.87]
Fazekas		PVWM	**0.82**	**0.79**	**0.74**	**0.81**	**0.78**	**0.72**	0.58	0.55	0.50
			[0.74 0.88]	[0.69 0.86]	[0.62 0.82]	[0.73 0.88]	[0.68 0.85]	[0.60 0.81]	[0.33 0.73]	[0.32 0.71]	[0.29 0.65]
		DWM	**0.68**	**0.66**	**0.62**	**0.67**	**0.65**	0.61	0.65	0.62	0.54
			[0.55 0.78]	[0.52 0.76]	[0.47 0.74]	[0.54 0.78]	[0.50 0.76]	[0.46 0.73]	[0.43 0.78]	[0.38 0.76]	[0.32 0.70]
		Tot	**0.81**	**0.80**	**0.77**	**0.80**	**0.79**	**0.76**	0.72	0.69	0.64
			[0.72 0.88]	[0.70 0.86]	[0.66 0.84]	[0.71 0.87]	[0.69 0.86]	[0.65 0.84]	[0.47 0.85]	[0.40 0.83]	[0.38 0.79]

The notation Pred4 indicates that the prediction was trained with the average of 4 raters. Ave3 indicates the comparison between the left out rater and the average of the three other raters. Bold font corresponds to results for which the prediction had a numerically higher ICC to the training average than the mean inter-rater variability with the average using the same number of raters. Underlined values reflect higher correlation of the prediction with the training average than the mean pairwise ICC (last column). For the scales, the partial total refers to the sum of the Scheltens subscales related to the periventricular (PV) and lobes while BG stands for basal ganglia. PV: periventricular; DWM: deep white matter; BGIT: basal ganglia and infratentorial region; IR: inter-rater. Pred4: prediction using the average of 4 raters; Pred3: prediction using the average of 3 raters; Pred2: prediction using the average of 2 raters; Ave3: comparison of 1 rater to the average of the 3 others; Ave2: comparison between 1 rater and the average of 2 others.

### Creation of an online training tool in WMH visual grading scales

**Fig. 6:**
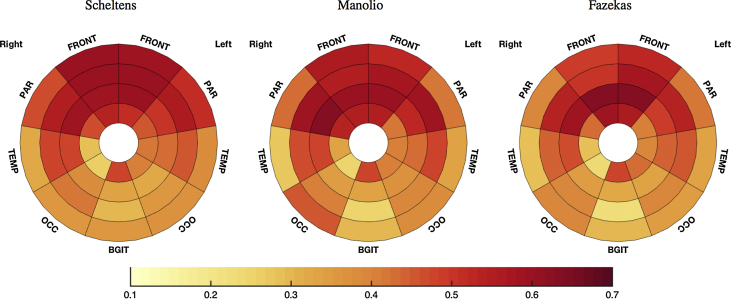
Screen-shot of the training system at the outset of the process to rate the periventricular subscales in the Scheltens scale. An explanation of the subscales description is always made available to the trainee.

With the recent advance in knowledge dissemination technologies, a web-based training suite was created to help improving the precision and accuracy of raters that is now available at (cmictig.cs.ucl.ac.uk/vrt/) For each of the twenty FLAIR scans of a training session, the participant can use an online viewer to scroll through the images and determine a score for each of the relevant subscales (cf. Fig. 6). After a training session is completed, color-coded regional performance metrics are provided through the bullseye representation, along with a textual interpretation of the training. This is to enable a local adjustment of the evaluation in a subsequent training.

## Discussion

We developed a novel regional-zonal analysis tool to represent WMH volume distribution and summarize it in a single bullseye infographic. We demonstrate the relevance of the new tool in deconstructing visual rating scales and evaluating rater performance, for which an online training tool for visual rating has been made available. Further applications may include comparison of populations, e.g. based on ethnicity, vascular risk factors or clinical mode of presentation.

The regional WMH burden features developed in this work were shown to characterize both spatial similarities and differences between visual rating scales, effectively deconstructing them.

The Manolio and the Fazekas scores showed similar spatial correlation patterns with an emphasis on the periventricular regions, while the Scheltens scores were shown to correlate in a more balanced fashion across brain regions. Our data-driven approach reveals the source of discrepancies between visual rating scores previously underlined [17], [21] with for instance the stronger impact of periventricular regions in the Manolio compared to the Scheltens scale. It can be used to better inform the choice of rating scales for a clinical study or to improve the implementation of rating protocols.

Secondly, our new tool can illustrate the spatial source of bias between a single rater and the consensus standard. We show that during the rating process, some readers paid more attention to a particular region than others. The regional maps reveal the anatomical locations that bias the rating behavior of a particular rater, which can be used to provide objective feedback.

Our model could therefore be used as a tool for training radiologists in order to improve their rating performance and calibrate the application of visual rating scales, reducing inter and intra-rater variability. Note that the presented maps estimate the per-region rater bias without modeling the associations between regions.

Thirdly, the regional loads were shown to be predictive of the local and global consensus rating scales. In order to test the ability to reproduce a consensus rating, both the automated algorithm and each human rater were compared to the consensus ratings. The automated prediction model performed similarly for most regions with a reduced variance, outperforming human raters for several regions.

Various factors can be put forward as limiting the model's ability to predict the consensus rating scores: first, an explicit choice was made regarding the regions relevant to each scale; second, the WMH burden feature used in this work (volume fraction) does not account for the size and count criteria of the Scheltens scale, a limitation that could be mitigated by including other local WMH features. The proposed predictive model performed better than human raters in subscales with a large degree of rater disagreement, possibly due to disagreements among raters with regards to the regional definitions [17].

One of the main strengths of this study is the number of raters involved in the visual grading of white matter hyperintensities in three different scales. This allows for an exhaustive comparison between raters and scales and an unbiased assessment of the utility of regional features and their ability to predict the average ratings. This study also has some limitations. The proposed method relies heavily on the accuracy of the automatic WMH segmentation and parcellation of the lobes, with segmentation errors directly impacting the analysis outcome. Also, due to ceiling and flooring effects in visual scale assessment, the correlation coefficient does not fully describe the relationship with regional WMH influence. Finally, the relevant regions used for feature extraction were selected empirically based on the literature descriptions, possibly affecting the ability to predict some outcomes.

The quality of clinical neuroimaging has continuously improved in the recent years, with the move to higher field strength (3T) and the use of more advanced sequences. For instance, the designs of the three visual rating scales mentioned in this study were based on 2D T2 spin echo or proton-density weighted images obtained on 1.5T or 0.35 T MR systems whereas clinical practice has evolved towards the use of T2 FLAIR imaging and volumetric data acquisition without slice gaps. With the known increase in sensitivity, specificity and correlation with clinical outcome when using 3T images [33], changes in rating scales are expected. At higher loads, the non-linear relationship between scores and volumes [19] contributes to a ceiling effect of the rating scales that may explain the high inter-rater correlation observed in this work compared to the literature [12]. In those cases, using volumes rather than scales appears more relevant and automated classification methods are therefore even more necessary.

## Conclusion

In conclusion, this work shows how the regional-zonal representation of WMH loads contributes to the deconstruction and comparison of visual rating scales, as well as the evaluation of raters. A web-based training suite has been made available (cmictig.cs.ucl.ac.uk/vrt/) that will expand the training potential of the local WMH assessment, aiming at helping the rater to perform local adjustments in their evaluation. Future work will evaluate the benefit obtained by using this training tool. Accurate semi-quantitative or quantitative assessments of WMH burden are likely to gain importance in the near future as WMH are biomarkers, which can be used for assessing disease progression, therapeutic intervention (such as blood pressure lowering drugs) or risk of intervention (carotid stenting). The bullseye plots will not only help train raters, but also visualize regional associations with risk factors or differences between populations.

## Acknowledgement and funding

Carole H. Sudre is funded by the Wolfson Foundation , UCL Faculty of EngineeringMRC (MR/M023664/1), EPSRC (EP/M020533/1), the NIHR Biomedical Research Centre (BRC345/NS/SB/101410) and Alzheimer's Society(AS-JF-17-011). Sebastien Ourselin receives funding from the EPSRC (EP/H046410/1, EP/J020990/1, EP/K005278), the MRC (MR/J01107X/1), the EU-FP7 project VPH-DARE@IT (FP7-ICT-2011-9-601055), the NIHR Biomedical Research Unit (Dementia) at UCL and the National Institute for Health Research University College London Hospitals Biomedical Research Centre (NIHR BRC UCLH/UCL High Impact Initiative-BW.mn.BRC10269). Ferran Prados is funded by the National Institute for Health Research College London Hospitals Biomedical Research Centre (NIHR BRC UCLH/UCL High Impact Initiative) and is a Guarantors of Brain fellow. Indran Davagnanam receives support from the NIHR UCLH/UCL BRC.

The Dementia Research Centre is supported by Alzheimer's Research UK, Brain Research Trust, and The Wolfson Foundation. M. Jorge Cardoso receives funding from EPSRC (EP/H046410/1). The SABRE study was funded at baseline by the UK Medical Research Council, Diabetes UK and the British Heart Foundation, and at follow-up by the Wellcome Trust (WT082464), British Heart Foundation (SP/07/001/23603 and CS/13/1/30327) and Diabetes UK (13/0004774).

## Disclosure of interest

The authors declare that they have no competing interest.
